# PROTOCOL: Parenting interventions to support parent/child attachment and psychosocial adjustment in foster and adoptive parents and children: A systematic review

**DOI:** 10.1002/cl2.1072

**Published:** 2020-01-31

**Authors:** Nina Thorup Dalgaard, Maiken Pontoppidan, Morten Kjær Thomsen, Bjørn Christian Arleth Viinholt, Trine Filges

**Affiliations:** ^1^ The Danish Center for Social Science Research VIVE Copenhagen Denmark

## Abstract

This is the protocol for a Campbell review. The objectives are as follows:

1. To assess the efficacy of attachment‐based interventions on measures of favourable parent/child outcomes (attachment security, dyadic interaction, parent/child psychosocial adjustment, behavioural and mental health problems and placement breakdown) within foster and adoptive families with children aged between 0 and 17 years.
2. To identify factors that appear to be associated with more effective outcomes and factors that modify intervention effectiveness (for example, age of the child at placement and at intervention start, programme duration, programme focus)

## 1 BACKGROUND

### 1.1 The problem, condition or issue

Every day, foster and adoptive parents around the globe care for children who have experienced serious adversity at a young age. From 2004 to 2010, between 29,000 and 45,000 children were internationally adopted each year (Selman, 2012). Globally, the number of intercountry adoptions is declining, and in 2013, there were three times fewer international adoptions worldwide than in 2003 (Mignot, 2015). However, domestic adoption out‐number intercountry adoption by far. Thus, the UN estimates that around 260,000 domestic adoptions take place worldwide each year^1^. In addition, it is estimated that approximately 2.7 million children worldwide are currently living in residential care arrangements. That is, in either foster or institutional care (Petrowski, Cappa & Gross, 2017).

Adopted children and children placed in foster care are at an increased risk of developing a range of mental health, behavioural and psychosocial adjustment problems (Bimmel, Juffer, van IJzendoorn, & Bakermans‐Kranenburg, 2003; Brand & Brinich, 1999; Oswald, Heil, & Goldbeck, 2010; Pears & Fisher, 2005; Pecora, Jensen, Romanelli, Jackson, & Ortiz, 2009). In a meta‐analysis based on 25,281 adoptees and 80,260 controls, adopted children showed more behaviour problems (effect sizes ranged between *d*, 0.16–0.24) and were much more likely to be referred for mental health services than their non‐adopted peers (*d*, 0.72). Furthermore, domestic adoptees showed more behaviour problems and were more likely to be referred for mental health services than international adoptees (Juffer & van IJzendoorn, 2005).

Foster children are also much more likely to experience behaviour and mental health problems than children living with their family of origin. Research on children placed in out‐of‐home care in the US suggests that between 23% and 61% of children under the age of 5 are significantly delayed when screened for developmental problems (Stahmer et al., 2005). Similarly, a study based on 267 children in foster care aged 0–17 years found the rate of behavioural problems in the clinical range to be two and a half time the expected rate in a comparable community sample (Clausen, Landsverk, Ganger, Chadwick, & Litrownik, 1998). Finally, a recent study by Turney & Wildeman (2016) based on data from the 2011–2012 US National Survey of Children's Health compared parent‐reported mental and physical health outcomes of children placed in foster care to outcomes of children not placed in foster care. In this study, Turney & Wildeman (2016) conclude that children in foster care are in poor mental and physical health relative to children in the general population. Thus, children placed in foster care were twice as likely to have learning disabilities, developmental delays, and speech problems. Furthermore, children placed in foster care were three times as likely to have ADD/ADHD, five times as likely to have anxiety, six times as likely to have behavioural problems, and seven times as likely to have depression (Turney & Wildeman, 2016). When studying the health of foster children it should, however, be noted, that there may potentially be discrepancies between countries due to differences in child protection legislature regulating at what point children are placed in foster care. Placing a child in foster care may be considered an intervention in itself, however, a recent meta‐analysis based on longitudinal studies of foster children showed that foster care does neither negatively nor positively influence the developmental trajectories of foster children, meaning that the mental health and behaviour problems in foster children when they entered foster care were unlikely to decrease over time (Goemans, van Geel, & Vedder, 2015). With regard to adopted children, longitudinal studies suggest a complex pattern of both vulnerabiliy and catch‐up, in which adopted children come to resemble their non‐adopted peers. In a consensus statement, researchers from different disciplines suggest that while there are significant benefits of adoption compared to remaining in vulnerable families or institutional care, adopted children are more vulnerable than non‐adopted children and some problems are likely to to persist postadoption (Palacios, Adrohor, et al., 2019). This evidence points to the need for interventions to support children in both foster and adoptive families.

Mental health issues and behaviour problems in foster and adopted children are often further exacerbated in the detrimental event of a placement breakdown (Goemans et al., 2015). Within research on adoption and foster care placement, different terminology has been employed to describe placement disruptions and breakdown. In this review, we use the term *placement breakdown* broadly to refer to the situation in which a foster or adopted child is either temporarily or permanently physically separated from the foster or adoptive parents regardless of the legal status. That is, whether the adoption process was finalised or not and whether the legal parental rights are terminated or not (Palacios, Rolock, Selwyn, & Barbosa‐Ducharne, 2019). Placement breakdown does, however, not refer to the situation in which a foster child is returned to the biological parents due to improvements in the parenting ability or the life circumstances of the biological parents. Nonetheless, an incidence of placement breakdown is difficult to estimate precisely due to variation in terminology, research designs, measurement, and available statistics. In a recent review of the existing literature on adoption breakdown and disruption, Palacios, Rolock, et al. (2019) report incidence rates ranging from just 1% to 23% in different studies. The study with the lowest incidence only measured disruptions or breakdown occurring in the timeframe when the adoption paper work was still being processed, which is not when most disruptions or breakdowns happen. In comparison, the study with the highest incidence was based on a population of children adopted at age 5–11 years. Age at adoption is known to be associated with placement breakdown, with older children being more at risk. These findings illustrate why the exact extend of the problem with adoption placement breakdown cannot be determined globally at this point. Similarly, it is estimated that between 20 and 50% of children in long term foster care will experience that their planned stay in their foster family ends prematurely (Oosterman, Schuengel, Slot, Bullens, & Doreleijers, 2007). Regardless of the exact extend of the problem with placement breakdown, it is clear that it constitutes a serious risk, as placement breakdowns are both costly to the society and can have devastating consequences for vulnerable children (Newton, Litrownik, & Landsverk, 2000; Palacios, Rolock, et al., 2019; Strijker, Knorth, & Knot‐Dickscheit, 2008).

In order to understand the aetiology of the problems experienced by foster and adoptive families and children, it has been suggested that the attachment relationship between the child and the adoptive/foster parents may be the origin of psychological vulnerability. John Bowlby's (1969) theory of attachment states that parent/child caregiving is a goal‐directed behavioural system accompanied by strong motivational effects and shaped by the adaptive function of protecting the offspring. Thus, through the child's interactions with the primary caregiver and based on the caregivers’ responses to the child's need of “a secure base”, inner working models of attachment are established early in life. Developing a secure attachment relationship with a parent or primary caregiver has long‐term benefits for children because of the impact on children's later adaptation and socio‐emotional development (Cassidy & Shaver, 2002). Ainsworth, Blehar, Waters, and Wall (1978) and Main and Solomon (1990) developed a typology of attachment patterns in children, which can be assessed and classified based on the child's behaviour in a laboratory exploration known as the Strange Situation Procedure. In children, attachment classification includes three categories describing organised attachment patterns; Secure, Insecure‐avoidant and Insecure‐resistant, as well as a fourth category known as Disorganised‐disoriented, which may be superimposed on the existing categorisation in cases where the child exhibits behaviours characterised by breakdowns of the organised attachment pattern. Securely attached children develop basic trust in self and others, enabling them to function autonomously. In the Strange Situation Procedure, securely attached toddlers are able to cope with a short separation from the caregiver and are easily comforted upon the return of the caregiver. In different ways, toddlers with insecure‐avoidant or insecure‐resistant attachment styles are less able to cope with the short separation from their primary caregiver, and they are not as easily comforted upon the return of their caregiver as the securely attached toddlers are. Some attachment scholars have proposed that toddlers in the avoidant category are frightened of appearing vulnerable. Thus, they may ignore their caregiver whilst still showing elevated signs of physiological arousal, indicating high levels of stress in response to the separation. Toddlers in the resistant category are hypothesised to perceive caregiving as unpredictable and thus may show a behaviour characterised by ambivalence towards the caregiver, such as excessive crying when the caregiver leaves and an inability of be comforted upon the return (Ainsworth et al.,^1978^). The Disorganised‐disoriented attachment category was added to the theory based on observations of children who did not seem to fit the description of the original patterns. Main and Solomon (1990) proposed that this category was characterised by breakdowns of attachment organisation following trauma. A number of studies support the associations between disorganised‐disoriented attachment in early childhood and the subsequent development of adverse child outcome (Alpern & Repacholi, 1993; Carlson, 1998; Groh et al., 2014; Moss et al., 2006).

Adopted children and children placed in foster care share experiences of early separation from caregivers, leaving them at elevated risk for developing insecure and/or disorganised‐disoriented attachment. In a series of meta‐analyses based on 39 studies (*n* = 2912 adopted children) Van den Dries, Juffer, van IJzendoorn, and Bakermans‐Kranenburg (2009) found that children, who were adopted after their first birthday, showed less attachment security than their non‐adopted peers did. This was similar to the attachment distributions in samples of children in foster care. Furthermore, Van den Dries and colleagues (2009) concluded that adopted children, regardless of age at the adoption and similarly to foster children, showed more disorganised attachment compared to their non‐adopted peers.

### 1.2 The intervention

In order to prevent placement breakdown and adverse child outcomes, a number of interventions are offered to foster and adoptive families. In this review, we will include attachment interventions aimed at helping the foster/adoptive children and their parents to form or sustain a secure attachment relationship. The interventions must be at least partly informed by attachment theory and aimed at enhancing parent/child attachment security and improving the psychosocial adjustment of parents and children by increasing parental sensitivity and emotional availability. Thus, in order to be included, an intervention must make explicit reference to attachment theory and treatment goals must include at least one of the following goals:

- Increased attachment security
- Decreased disorganised attachment
- Increased parental sensitivity
- Increased parental emotional availability
- Increased positive dyadic interaction
- Increased psychosocial adjustment of the child

Examples of eligible interventions are: ***Attachment and Biobehavioral Catch‐up (ABC), Video‐Feedback Intervention to Promote Positive Parenting (VIPP), Parent‐Child Interaction Therapy (PCIT), and The Circle of Security (COS).*** ABC is a manualized intervention consisting of 10 weekly 60‐min sessions delivered in the families’ homes. Each session is videotaped and used for both clinical supervision of the therapists and video‐feedback for parents (Yarger, Bernard, Caron, Wallin, & Dozier, 2019). VIPP is a video‐feedback short‐term home‐based intervention, which exists in several different versions with and without a component that adresses the parental attachment representations and with an additional intervention module which seeks to promote sensitive discipline (Juffer, Bakermans‐Kranenburg, & van IJzendoorn, 2008). There are several different COS protocols, but key components of the intervention include an individualised assessment of the parent–child relationship. Later sessions involve feedback to parents regarding their interaction with the child as well as education about attachment and an opportunity to reflect on their child's specific needs and the challenges faced by each family (Zeanah, Berlin, & Boris, 2011; Woodhouse, Powell, Cooper, Hoffman, & Cassidy, 2018). PCIT is only partly informed by attachment theory. PCIT is a 14‐ to 20‐week manualised intervention founded on social learning, behavioural, and attachment theories. The intervention takes place in a clinical setting in which therapists coach parents from an observation room behind a two‐way mirror via a bug‐in‐the‐ear‐receiver (Allen, Timmer, & Urquiza, 2014).

We will exclude interventions that do not involve the direct participation of at least one parent and a foster/adopted child in at least one session. Thus, we will exclude individual parental or couples’ therapy, parental counselling, psychoeducation, and individual child psychotherapy or adult/child support groups. Furthermore, we will exclude interventions which are not based on attachment theory, such as cognitive behaviour therapy or trauma‐focused therapies.

Finally, we will exclude interventions, which are deemed unvalidated or theoretically questionable such as interventions claiming to promote “reattachment” through coercive holding, physical restraints, or rebirthing (Chaffin et al., 2006). Unvalidated treatments refer to treatments with serious harmful side effects, treatments with no empirical evidence to support their claim of effectiveness, treatments based on ideas which fails to mesh with current accepted theory, treatments which are not discussed in professional publications such as peer reviewed journals, and treatments based exclusively on clinical observations rather than science (see Mercer, Sarner, & Rosa, 2003 for a full description of criteria to determine if a treatment is unvalidated). In this review, this means that we will exclude interventions that involve physically forcing a child to submit to being held or to sustain eye contact or to promote regression to achieve “reattachment” or to “vent anger” while being restrained. These interventions are excluded as they are not only contraindicated but have led to injury and even death (Mercer et al. 2003; Zeanah et al., 2011).

**How the intervention might work** “Although the capacity for developmental change diminishes with age, change continues throughout the life cycle so that changes for better or for worse are always possible. It is this continuing potential for change that means that at no time of life is a person invulnerable to every possible adversity and also at no time of life is a person impermeable to favourable influence. It is this persisting potential for change that gives opportunity for effective therapy” (Bowlby, 1988, p. 154)

By definition, foster and adopted children have experienced a separation from their biological parents. Furthermore, foster and adopted children have often experienced adverse events such as insufficient medical care, malnutrition, physical and emotional abuse, and neglect prior to their initial placement (Sullivan & van Zyl, 2008). Thus, the children may be physically and psychologically vulnerable at the time of the placement and this vulnerability may continue to manifest itself throughout their life course in various ways (Palacios, Rolock, et al., 2019). However, as proposed by Bowlby within the above quotation, attachment is a dynamic phenomenon, and there is continuous potential for positive changes through the child's interactions with sensitive caregivers. This potential is what attachment interventions are aimed at supporting. According to Bowlby (1988), a child will experience grief, anger and distress as a result of temporary or permanent loss of access to existing attachment figures, and this can only be resolved if the child is able to develop new attachment relationships with alternative caregivers. By supporting a child's current caregivers (eg. the foster or adoptive parents) in meeting the needs of the child in a consistent and sensitive manner, attachment interventions are proposed to be able to change the child's internal working models of attachment, leading to an increased sense of “felt security”. Attachment interventions are thus designed to help foster and adoptive parents to notice and understanding subtle and overt emotional cues in their child's behaviour and to respond to these cues in a sensitive, contingent, and consistent manner. Sometimes this process may involve a reflection on the attachment history and the current “states of mind” with respect to attachment of the adoptive or foster parents themselves (Juffer et al., 2008). The reason for the assumed benefits of working with the caregiver's own state of mind with respect to attachment is that this has been identified as the strongest predictor of whether foster children will become securely attached to a foster parent or not (Dozier, Stoval, Albus, & Bates, 2001). However, in a study comparing VIPP and VIPP‐R with a control condition in a sample consisting of mothers with less than 14 years of education, who were classified as insecure and their first born infants, Veldenman, Bakermans‐Kranenburg, Juffer, and van IJzendorn (2006) found overall differences between the two intervention groups and the control group on meassures of maternal sensitivity post‐intervention, but no significant differences were found between the two interventions, suggesting no added benefits of working with maternal attachment representations compaired to the VIPP intervention alone.

Typically, attachment interventions consist of sessions in which the therapist is working with the parent(s) and children simultaneously. In subsequent sessions, the parents are provided with feedback, education about attachment, and are invited to reflect upon their experiences with the child.

### 1.3 Why it is important to do this review

Based on findings on the associations between early disorganised‐disoriented attachment and subsequent adverse child outcomes, a number of interventions and programs are aimed at supporting the development of a secure attachment relationship between parents and children (Dozier & Rutter, 2008; Dozier et al., 2001). In a meta‐analysis on the effectiveness of preventative attachment interventions on parental sensitivity and infant attachment for at risk populations (*k* = 70), results suggest that interventions are rather effective at increasing parental sensitivity (*d* = 0.20) and/or infant attachment (*d* = 0.30; Bakermans‐Kranenburg, Van IJzendoorn, & Juffer, 2003). In a systematic review co‐registered at Cohrane and Campbell, Barlow, Bennett, Midgley, Larkin, and Wei (2015) explored the efficacy of attachment‐based parent–infant psychotherapy on parental and infant mental health. This review focused on infants aged 0–24 months within vulnerable families (defined as families in which parents were suffering from mental health issues, drug/alcohol abuse, or were victims of domestic violence). Findings from this review suggest that parent–infant psychotherapy is a promising approach in terms of improving infant attachment security in high‐risk families. However, there were no significant differences compared with no treatment or treatment‐as‐usual for other parent‐based or relationship‐based outcomes, and no evidence that parent–infant psychotherapy is more effective than other ways of working with parents and infants. The review by Barlow and colleagues provides important insight into the efficacy of attachment‐based interventions, however, the findings may not be applicable to the population of the present review for two main reasons. First, the population of adoptive and foster parents are typically resourceful individuals highly motivated for participation in interventions. Second, Barlow, Bennett, Midgley, Larkin, and Wei (2015) focused only on therapeutic interventions that could be described as parent–child psychotherapy and only included infants under the age of 2 years. Juffer, Bakermans‐Kranenburg, and van IJzendoorn (2017) provides a review and meta‐analysis of the effectiveness of Video‐feedback Intervention to promote Positive Parenting and Sensitive Discipline (VIPP‐SD), which is an intervention based partially on attachment theory with various populations of at‐risk parents and vulnerable children (*k* = 12). In the review, positive effects of VIPP‐SD were found on measures of sensitive parenting and socio‐emotional child outcomes.

Kerr and Cossar (2014) conducted a systematic review of studies of attachment interventions with foster and adoptive parents and children aged 0–17 years. This narrative review provides preliminary insights and suggests that there are positive effects of attachment interventions for this population, but it lacks the methodological rigour of a Campbell review and needs to be updated. Furthermore, the review by Kerr and Cossar (2014) only provides a very limited description of the quality appraisal process and it does not include meta‐analysis. Thus, the efficacy of attachment interventions on measures of both attachment security and on measures of parent/child psychosocial adjustment in foster and adoptive families is yet to be thoroughly examined, which is where the present review will contribute.

Finally, a recent meta‐analytic review (*k* = 53) examined the effects of all types of parenting interventions in foster care and adoption on eight types of outcomes (Schoemaker et al., 2019). Results show positive effects on four parenting outcomes (sensitive parenting, dysfunctional discipline, parenting knowledge and attitudes and parenting stress, and on one child outcome (behaviour problems), whereas the review didn't find effects for attachment security, child diurnal cortisol levels or placement disruptions. This review provides many insights, however the present review will provide an extensive risk of bias analysis of each included study, update the searches and focus exclusively on the specific effectiveness of attachment‐based interventions.

The Hague Convention of 29 May 1993 on Protection of Children and Co‐operation in Respect of Intercountry Adoption (Hague Adoption Convention) requires all signatory states to: *“promote the development of adoption counselling and post‐adoption services in their States”* (article 9). In Denmark, this has resulted in the 2016 establishment of national post‐adoption services providing all adoptive parents with post‐adoption family counselling. The present review will provide knowledge on the efficacy of a range of interventions commonly offered to adoptive parents. Furthermore, the present review is relevant for child protection agencies across the world with the authority to place children in foster care.

## 2 OBJECTIVES

1. To assess the efficacy of attachment‐based interventions on measures of favourable parent/child outcomes (attachment security, dyadic interaction, parent/child psychosocial adjustment, behavioural and mental health problems and placement breakdown) within foster and adoptive families with children aged between 0 and 17 years.
2. To identify factors that appear to be associated with more effective outcomes and factors that modify intervention effectiveness (for example, age of the child at placement and at intervention start, programme duration, programme focus)

## 3 METHODS

### 3.1 Criteria for considering studies for this review

#### 3.1.1 Types of studies

In order to summarise what is known about the causal effects of attachment interventions on parent/child attachment and children's psychosocial adjustment, we will include all studies with a well‐defined control group. Thus, the study designs eligible for inclusion are:

1. Controlled trials

- Randomised controlled trials (RCTs)
- Quasi‐randomised controlled trial designs (QRCTs). Here participants are allocated by means, which are not expected to influence outcomes, for example alternate allocation, participant's birth data, case number or alphabetic order.

2. Quasi‐experimental studies (QES), This category refers to both studies, where participants are allocated by other actions controlled by the researcher, or where allocation to the intervention and control group are not controlled by the researcher (for example by time differences or policy rules). In order to be included, QESs must credibly demonstrate that outcome differences between intervention and control groups are the effect of the intervention and not the result of systematic baseline differences between groups. That is, selection bias should not be driving the results. This assessment is included as part of the risk of bias tool, which we elaborate on in Section 4.6.4.

3. We will include studies without a control group only if they measure attachment as categorical data preintervention and postintervention and compare the findings with a distribution of categories in a relevant large‐scale normative non‐clinical sample. The reason for this is that the purpose of attachment interventions is to promote a catch‐up among adoptees/foster children post intervention. By catch‐up, we mean a situation in which the distribution of attachment categories post intervention resembles that of a normative nonclinical sample (Van den Dries et al., 2009). If included, these studies will be analysed separately.

Studies using single group pre‐post comparisons will not be included.

#### 3.1.2 Types of participants

We will include foster and adoptive families (both single and two‐parent families) with at least one child aged between 0 and 17 years at the beginning of the intervention. Families must be residents in an OECD country.

#### 3.1.3 Types of interventions

We will include attachment interventions with foster and adoptive parents in any setting (i.e., clinic, hospital, or home) and in any format (i.e., family or multi‐family therapy). Interventions must be at least partly based on attachment theory and aimed at enhancing parent/child attachment security and improving the psychosocial adjustment of parents and children by increasing parental sensitivity and emotional availability.

We will exclude interventions that do not involve the direct participation of at least one parent and a foster/adopted child (such as individual or couples’ therapy, parental counselling, psychoeducation).

Comparison can consist of no treatment, treatment as usual/other interventions/treatments offered (including normal service provision), or wait‐list control.

Effect sizes from comparison studies in which two alternative interventions are compaired against each other are not fully comparable to effect sizes from treatment‐control designs. We therefore plan to analyse two‐treatment comparison designs separately from treatment‐control designs. If two‐treatment comparison design effect sizes cannot be pooled, study‐level effects will be reported narratively.

#### 3.1.4 Types of outcome measures

We will extract the following outcomes if they are assessed using measures previously validated on other samples than the intervention sample (parent‐report or independent observation). We list examples of measures for each outcome. Timing of outcome assessments will include immediately post‐intervention and follow‐up time points.

##### Parent outcomes

Parental mental health/psychosocial adjustment. For example: depression, Beck Depression Inventory (BDI; Beck et al., 1961), anxiety, Beck Anxiety Inventory (BAI; Beck et al., 1988), parenting stress, Parenting Stress Index (PSI; Abidin, 1983).

Parental Attachment. For example: the Adult Attachment Interview (Main & Goldwyn, 1994)

Parental reflective function. For example: Parent Development Interview – PDI (Slade et al., 2004), and the Parental Reflective Functioning Questionnaire (Luyten, Mayes, Nijssens, & Fonagy, 2017)

##### Parent–child relationship outcomes

Parent–Child interaction. For example: CARE‐Index (Crittenden, 2001), Emotional Availability Scales (EAS; Biringen et al., 1993), Dyadic Parent‐Child Interaction Coding System (DPICS; Robinson and Eyberg, 1981)

Parental sensitivity. For example: Maternal Sensitivity Scale (Ainsworth et al., 1974), or Frightened/Frightening (FR) Coding System Frightened/Frightening (FR) Coding System (Main & Hesse, 1992)

##### Child outcomes

Child psychosocial adjustment. For example: Eyberg Child Behaviour Inventory (ECBI; Eyberg and Ross, 1978), the Behaviour Screening Questionnaire (BSQ; Richman and Graham, 1971), the Child Behaviour Questionnaire (CBQ; Rutter et al., 1970), Infant and Toddler Social and Emotional Adjustment Scale – ITSEA (Carter and Briggs‐Gowan, 2000)

Child attachment security; for example, Strange Situation Procedure (SSP; Ainsworth et al., 1971), Preschool Measure of Attachment (Crittenden, 1992)

##### Adverse outcomes

Any adverse effects of interventions will be included as an outcome including a worsening of outcome on any of the included measures.

Time points for measures considered will be:

- Post‐intervention
- 0 to 1 year follow up
- 1 to 2 year follow up
- More than 2 year follow up

Follow‐up at any given point in time will be included if meaningful based on the objectives of the review. That is, if possible, we will include follow‐up data regarding placement breakdown, contacts with child protection, and post adoption services during the remainder of the children's childhoods as well as contacts with psychiatric services throughout the children's life course.

### 3.2 Primary outcomes

Due to the objectives of the present review, we do not distinguish between primary and secondary outcomes.

### 3.3 Secondary outcomes

Due to the objectives of the present review, we do not distinguish between primary and secondary outcomes.

### 3.4 Types of settings

The review will include attachment interventions with foster and adoptive families in any setting. Hence, the intervention may take place in the families’ homes, in an outpatient clinic, or hospital or in community‐based facilities. Furthermore, we will include interventions delivered in any format. That is, we will include interventions that are delivered to both individual families or dyads or to multiple families at a time. We will exclude interventions that do not involve face‐to‐face interaction between participants and therapists.

### 3.5 Search methods for identification of studies

#### 3.5.1 Search strategy

Relevant studies will be identified through searches in electronic databases, grey literature repositories and resources, hand searches in specific targeted journals, citation tracking, contact to international experts, and internet search engines. Following bibliographic databases will be searched:

- SocINDEX
- PsycINFO
- EconLit
- ERIC
- CINAHL
- Academic Search
- Science Citation Index
- Social Science Citation Index
- Sociological Abstracts

#### 3.5.2 Electronic searches

Below is an example of search strategy, as it will be conducted on the SocIndex database. Whenever adequate and possible, the conducted searches will include subject heading searches from the thesaurus of the respective database.

**Table:** 

S15	S5 AND S8 AND S11 AND S14
S14	S12 OR S13
S13	AB (treatmen* OR intervent* OR therap* OR program*)
S12	TI (treatmen* OR intervent* OR therap* OR program*)
S11	S9 OR S10
S10	AB (effect* OR trial* OR experiment* OR control* OR random* OR impact* OR compar*)
S9	TI (effect* OR trial* OR experiment* OR control* OR random* OR impact* OR compar*)
S8	S6 OR S7
S7	AB (sensitiv* OR emoti* OR dyadic* OR attach* OR relation*)
S6	TI (sensitiv* OR emoti* OR dyadic* OR attach* OR relation*)
S5	S1 OR S2 OR S3 OR S4
S4	TI out‐of‐home OR AB out‐of‐home N6 (care* OR place*)
S3	AB (adopt* OR foster*) N6 (parent* OR child* OR famil* OR home*)
S2	TI (adopt* OR foster*) AND TI (parent* OR child* OR famil* OR home*)
S1	TI foster care* OR AB foster care*

#### 3.5.3 Searching other resources

We will search specifically after three types of grey literature: working papers, reports and dissertations. Some of the bibliographic databases also cover grey literature (ERIC per example). We will search the following resources for grey literature:

- ProQuest Dissertations & Theses Global (dissertations) (EBSCO‐host)
- EBSCO Open Dissertations (dissertations) (EBSCO‐host)
- Open Grey (reports, working papers, dissertations) ‐ http://www.opengrey.eu/
- Google Scholar (reports, working papers, dissertations) ‐ https://scholar.google.com/
- Google searches (reports, working papers, dissertations) ‐ https://www.google.com/
- Social Care Online (reports, working papers, dissertations, systematic reviews) ‐ https://www.scie‐socialcareonline.org.uk/
- Social Science Research Network (working papers) ‐ https://www.ssrn.com/index.cfm/en/
- Danish National Research Database (working papers, articles, dissertations, systematic reviews) ‐ https://www.forskningsdatabasen.dk/en
- SocArXiv

Further resources for identifying grey literature may be added during the search process. A final list of grey literature resources will be included in the appendix of the review

##### Hand search

4 specific journals will be hand‐searched:

Attachment & Human Development

Adoption & Fostering

Adoption Quarterly

Children and Youth Services Review

##### Citation tracking

In order to identify both published studies and grey literature, we will utilise citation‐tracking/snowballing strategies. Our primary strategy will be to citation‐track related systematic‐reviews and meta‐analyses. The review team will also check reference lists of included primary studies for new leads.

##### Contact with international experts

We will contact international experts to identify unpublished and ongoing studies, and provide them with the inclusion criteria for the review along with the list of included studies, asking for any other published, unpublished or ongoing studies relevant to the review. We will primarily contact corresponding authors of the related reviews mentioned in the Prior reviews section, but contacts will be extended to others if we find references to or mentions of ongoing studies in screened publications.

### 3.6 Data collection and analysis

#### 3.6.1 Description of methods used in primary research

We expect that a certain amount of the included studies will be conducted without randomisation of participants. In order to be eligible for inclusion, studies comparing two groups of adoptees/foster children must adequately deal with between‐group differences on all relevant variables at baseline (i.e., basic sociodemographic variables, age of adopted/foster child both at the time of the intervention, and adoption/beginning of foster care as well as on child outcome measures). The methodological appropriateness will be assessed according to the risk of bias model outlined in section “*Assessment of risk of bias in included studies*.” The risk of bias assessment makes it possible to discriminate between studies with varying degrees of risks. Studies that have been coded with a *Critical risk of bias* will not be included in the data synthesis. An example of a study that may be included is Yarger et al. (2019). In this study, 120 internationally adopted children aged between 6.8 months and 48.4 months (M = 21.9 months, *SD* = 9.0 months) and their adoptive parents were randomly assigned to receive either the Attachment and Biobehavioral Catch‐up intervention or a control intervention. Outcomes included parental sensitivity (i.e., contingent responsiveness to child's cues), parental intrusiveness (i.e., physical and/or verbal behaviour that interferes with the child's autonomy), and parental positive regard (i.e., positive affect expressed towards the child) and these were measured preintervention and postintervention and at annual follow‐up visits 1 and 2 years after the intervention. Another example of a study, which may be included is Barone, Ozturk, and Lionetti (2018). In this study, 83 post‐institutionalised children and their adoptive mothers were randomised to receive either the Video‐Feedback Intervention to promote Positive Parenting (VIPP) or a dummy intervention. Outcomes included maternal emotional availabilty and child behaviour problems.

#### 3.6.2 Selection of studies

First, under the supervision of review authors, two team assistants will independently screen titles and abstracts to exclude studies that are clearly irrelevant. Studies considered eligible by at least one assistant or studies in which there is insufficient information in the title and abstract to judge eligibility, will be retrieved in full text. The full texts will then be screened independently by two review team assistants under the supervision of the review authors. Any disagreement of eligibility will be resolved by the review authors. Exclusion reasons for studies that otherwise might be expected to be eligible will be documented and presented in an appendix.

The study inclusion criteria will be piloted by the review authors (see Appendix A). The overall search and screening process will be illustrated in a flow diagram. None of the review authors will be blind to the authors, institutions or the journals responsible for the publication of the articles.

#### 3.6.3 Data extraction and management

Two review authors will independently code and extract data from included studies. A coding sheet will be piloted on several studies and revised as necessary (see *Data extraction*, Appendix A). Disagreements will be resolved by consulting a third review author with extensive content and methods expertise. Disagreements resolved by a third reviewer will be reported. Data and information will be extracted on available characteristics of participants, intervention characteristics and control conditions, research design, sample size, risk of bias and potential confounding factors, outcomes and results. Extracted data will be stored electronically.

#### 3.6.4 Assessment of risk of bias in included studies

We will assess the risk of bias in randomised studies using Cochranes revised risk of bias tool, ROB 2 (Higgins et al., 2019).

The tool is structured into five domains, each with a set of signalling questions to be answered for a specific outcome. The five domains cover all types of bias that can affect results of randomised trials.

The five domains for individually randomised trials are:

1. Bias arising from the randomisation process;
2. Bias due to deviations from intended interventions (separate signalling questions for effect of assignment and adhering to intervention);
3. Bias due to missing outcome data;
4. Bias in measurement of the outcome;
5. Bias in selection of the reported result.

We do not expect to include cluster‐randomised trials, but if we do, an additional domain is included ([1b] Bias arising from identification or recruitment of individual participants within clusters). We will use the latest template for completion (currently it is the version of 15 March 2019 for individually randomised parallel‐group trials and 20 October 2016 for cluster randomised parallel‐group trials). In the cluster randomised template, however, only the risk of bias due to deviation from the intended intervention (effect of assignment to intervention; intention to treat ITT) is present and the signalling question concerning the appropriateness of the analysis used to estimate the effect is missing. Therefore, for cluster randomised trials, we will only use the signalling questions concerning the bias arising from identification or recruitment of individual participants within clusters from the template for cluster randomised parallel‐group trials; otherwise we will use the template and signalling questions for individually randomised parallel‐group trials.

We will assess the risk of bias in non‐randomised studies, using the model ROBINS–I, developed by members of the Cochrane Bias Methods Group and the Cochrane Non‐Randomised Studies Methods Group (Sterne et al., 2016a). We will use the latest template for completion (currently it is the version of 19 September 2016).

The ROBINS‐I tool is based on the Cochrane RoB tool for randomised trials, which was launched in 2008 and modified in 2011 (Higgins and Green, 2011).

The ROBINS‐I tool covers seven domains (each with a set of signalling questions to be answered for a specific outcome) through which bias might be introduced into non‐randomised studies:

1. Bias due to confounding
2. Bias in selection of participants
3. Bias in classification of interventions
4. Bias due to deviations from intended interventions;
5. Bias due to missing outcome data;
6. Bias in measurement of the outcome;
7. Bias in selection of the reported result.

The first two domains address issues before the start of the interventions and the third domain addresses classification of the interventions themselves. The last four domains address issues after the start of interventions and there is substantial overlap for these four domains between bias in randomised studies and bias in non‐randomised studies trials (although signalling questions are somewhat different in several places, see Sterne et al. (2016) and Higgins et al. (2019).

Randomised study outcomes are rated on a “Low/Some concerns/High” scale on each domain; whereas non‐randomised study outcomes are rated on a “Low/Moderate/Serious/Critical/No Information” scale on each domain. The level “Critical” means: the study (outcome) is too problematic in this domain to provide any useful evidence on the effects of intervention and it is excluded from the data synthesis. The same critical level of risk of bias (excluding the result from the data synthesis) is not directly present in the RoB 2 tool, according to the guidance to the tool (Higgins et al., 2019).

We will add a critical level of risk of bias to the RoB 2 tool with the same meaning as in the ROBINS‐I tool; that is, the study (outcome) is too problematic in this domain to provide any useful evidence on the effects of intervention and it is excluded from the data synthesis. We will stop the assessment of a randomised study outcome using the RoB 2 as soon as one domain is judged as “Critical.” Likewise, we will stop the assessment of a non‐randomised study outcome as soon as one domain in the ROBINS‐I is judged as “Critical.”

“High” risk of bias in multiple domains in the RoB 2 assessment tool may lead to a decision of an overall judgement of “Critical” risk of bias for that outcome and it will be excluded from the data synthesis. “Serious” risk of bias in multiple domains in the ROBINS‐I assessment tool may lead to a decision of an overall judgement of “Critical” risk of bias for that outcome and it will be excluded from the data synthesis.

##### Confounding

An important part of the risk of bias assessment of non‐randomised studies is consideration of how the studies deal with confounding factors. Systematic baseline differences between groups can compromise comparability between groups. Baseline differences can be observable (e.g., age and gender) and unobservable (to the researcher; e.g., motivation and “ability”). There is no single non‐randomised study design that always solves the selection problem. Different designs represent different approaches to dealing with selection problems under different assumptions, and consequently require different types of data. There can be particularly great variations in how different designs deal with selection on unobservables. The “adequate” method depends on the model generating participation, that is, assumptions about the nature of the process by which participants are selected into a programme.

A major difficulty in estimating causal effects of attachment interventions on child outcomes is the potential heterogeneity in the children's developmental histories. Some children have suffered extreme abuse and neglect prior to being placed in foster care or adopted and information about the child's experiences prior to the placement/adoption may not be available to the foster/adoptive parents or to the researcher. Children who have experienced physical, emotional and/or sexual abuse may not present with symptoms straight away, as there is not a straight forward causal relationship between traumatic experiences and measurable psychopathological post‐traumatic symptoms. Sometimes a child may experience trauma and appear resilient at first but begin to show symptoms many years after the traumatic experiences.

Thus, differences in the children's mental health and psychosocial adjustment may appear insignificant at baseline but could potentially be an unobservable source of bias.

As there is no universal correct way to construct counterfactuals for non‐randomised designs, we will look for evidence that identification is achieved, and that the authors of the primary studies justify their choice of method in a convincing manner by discussing the assumption(s) leading to identification (the assumption(s) that make it possible to identify the counterfactual). Preferably the authors should make an effort to justify their choice of method and convince the reader that the only difference between a treated child and a non‐treated child is the treatment. The judgement is reflected in the assessment of the confounder unobservables in the list of confounders considered important at the outset (see *User guide for unobservables*, Appendix A).

In addition to unobservables, we have identified the following observable confounding factors to be most relevant: Age at placement/adoption and at the intervention, children's history of trauma prior to placement/adoption, country of origin, and socioeconomic background of foster/adoptive parents.

##### Importance of pre‐specified confounding factors

The motivation for focusing on age at placement/adoption and at the intervention, children's history of trauma prior to placement/adoption, country of origin, and socioeconomic background of foster/adoptive parents is given below.

Children's age at placement/adoption is known to be associated with successful placement and adoption, with older children being at a higher risk of insecure attachment, behavioural problems, and placement breakdown (Oosterman et al., 2006; Palacios, Rolock, et al., 2019; Van den Dries et al., 2009). Attachment interventions are most often designed to target younger children, and thus the suitablility of these interventions for older children is less well established (Juffer, Bakermans‐Kranenburg, & van IJzendoorn, 2008). Therefore, to be sure that an effect estimate is a result from a comparison of groups with no systematic baseline differences, it is important to control for the children's age both at placement/adoption and at the intervention. For the reasons specified above, it is important to control for children's history of abuse and neglect prior to adoption or placement, as traumatic experiences may influence children's later developmental trajectories in a multitude of ways. We are aware, however, that in some cases the children's history of abuse and neglect will not be available to the researchers and in this case the study may still be included if there is nothing to suggest systematic differences in child abuse histories.

Specifically for adopted children, it is important to control for the country of origin, as previous research has documented systematic differences between domestic and international adoptees on measures of mental health (Juffer & van IJzendoorn, 2005). Furthermore, a study comparing children adopted from US foster care, US private agencies and internationallly adopted children found significant differences in mental health service utilazation use (Tan & Marn, 2013). For international adoptees, systematic differences have been found between different countries of origin, with children from countries of origin such as Romania being more at risk for later maladaptation (Marcovitch, Cesaroni, Roberts, & Swanson, 1995), whereas children adopted from China have been found to have a significantly higher parent/child relationship quality (Tan, Major, Marn, Na, & Jackson, 2015). A large body of research documents the impact of parental socioeconomic background on almost all aspects of children's development (Renninger & Sigel, 2006), which is why we consider it important to control for this. Socioeconomic background factors are, for example, adoptive/foster parents’ educational level, family income, minority background, etc. A study by Tiemans, van der Ende, and Verhulst (2005) based on a sample of 1484 young internationnal adoptees (aged 10–15 years) and 695 nonadopted controls in the Netherlands found that for all psychiatric diagnoses together internationally adopted children from low and middle parental economic backgrounds did not differ from comparison subjects, however internationally adopted children in families with high parental socioeconomic status were 2.17 times more likely to meet the criteria for a disorder as nonadoptess from families with high parental socioeconomic status.

##### Effect of primary interest and important co‐interventions

We are mainly interested in the effect of participating in and completion of the intended intervention, that is, the treatment on the treated effect. The risk of bias assessments will therefore be in relation to this specific effect. The risk of bias assessments of both randomised trials and non‐randomised studies will consider adherence and differences in additional interventions (“co‐interventions”) between intervention groups. Important co‐interventions will be the regular support systems available to foster/adoptive families after placement/adoption of the child. This may include parents counselling, education, or informal support networks throughout the children's time in their care.

##### Assessment

At least two review authors will independently assess the risk of bias for each relevant outcome from the included studies. Any disagreements will be resolved by a third reviewer with content and statistical expertise and will be reported. We will report the risk of bias assessment in risk of bias tables for each included study outcome in the completed review.

#### 3.6.5 Measures of treatment effect

##### Continuous outcomes

For continuous outcomes, effects sizes with 95% confidence intervals will be calculated, where means and standard deviations are available. If means and standard deviations are not available, we will calculate standardised mean differences (SMDs) from *F*‐ratios, *t*‐values, *χ* ^2^ values and correlation coefficients, where available, using the methods suggested by Lipsey & Wilson (2001). If not enough information is yielded, the review authors will request this information from the principal investigators. Hedges’ *g* will be used for estimating SMDS. Any standardised measures of children's development, psychosocial adjustment and mental health are examples of relevant continuous outcomes in this review.

##### Dichotomous outcomes

For dichotomous outcomes, we will calculate odds ratios with 95% confidence intervals. Placement breakdown or not is an example of a relevant dichotomous outcome in this review. If we include primary studies using a categorical measure of attachment as an outcome, we will create multiple dichotomous variables such as secure versus insecure, disorganised verus organised to calculate effect sizes as odds ratios.

There are statistical approaches available to re‐express dichotomous and continuous data to be pooled together (Sánchez‐Meca, Marín‐Martínes & Chacón‐Moscoso, 2003). In order to calculate common metric, odds ratios will be converted to SMD effect sizes using the Cox transformation. We will only transform dichotomous effect sizes to SMD, if appropriate, for example, as may be the case with for example the outcomes attachment security that can be measured with both binary and continuous data. When effect sizes cannot be pooled, study‐level effects will be reported in as much detail as possible. Software for storing data and statistical analyses will be RevMan 5.0, Excel, R, and Stata 10.0.

#### 3.6.6 Unit of analysis issues

We will take into account the unit of analysis of the studies to determine whether individuals were randomised in groups (i.e., cluster‐randomised trials), whether individuals may have undergone multiple interventions, whether there were multiple treatment groups, and whether several studies are based on the same data source.

##### Cluster randomised trials

The randomisation of clusters can result in an overestimate of the precision of the results (with a higher risk of a Type I error) where their use has not been compensated for in the analysis. In the unlikely event that we include cluster RCTs, the impact of the inclusion of data from such studies in the meta‐analyses will be explored using a sensitivity analysis and any necessary adjustments to the data will be made, using available estimates of ICC.

##### Multiple interventions groups and multiple interventions per individuals

We are unlikely to identify cross‐over studies as the effects of therapy are intended to be long term. Therefore, cross‐over from a treatment condition to no‐treatment condition would not be feasible. For studies with more than one active intervention and only one control group, we will select the intervention that most closely matches our inclusion criteria and will exclude the other(s) (Higgins & Green, 2011).

##### Multiple studies using the same sample of data

In some cases, several studies may have used the same sample of data or some studies may have used only a subset of a sample used in another study. We will review all such studies, but in the meta‐analysis, we will only include one estimate of the effect for each conceptual outcome from each sample of data. This is done to avoid dependencies between the “observations” (i.e., the estimates of the effect) in the meta‐analysis. The choice of which estimate(s) to include will be based on our risk of bias assessment of the studies. If there are multiple estimates of effects regarding the same outcome (such as child mental health), we will choose the estimate from the study that we judge to have the least risk of bias (primarily, confounding bias). If two (or more) studies are judged to have the same risk of bias and one (or more) of the studies uses a subset of a sample used in another study (or studies), we will include the study using the full set of participants.

##### Multiple time points

When the results are measured at multiple time points, each outcome at each time point will be analysed in a separate meta‐analysis with other comparable studies taking measurements at a similar time point. As a general guideline, these will be grouped together as follows: (a) post‐intervention, (b) 0 to 1 year follow up, (c) 1 to 2 year follow up, and (d) More than 2 year follow up. However, should the studies provide viable reasons for an adjusted choice of relevant and meaningful duration intervals for the analysis of outcomes, we will adjust the grouping.

#### 3.6.7 Dealing with missing data

Missing data and attrition rates in the individual studies will be assessed using the risk of bias tool. Studies must permit calculation of a numeric effect size for the outcomes to be eligible for inclusion in the meta‐analysis. Where studies have missing summary data, such as missing standard deviations, we will derive these where possible from, for example, *F*‐ratios, *t*‐values, *χ* ^2^ values and correlation coefficients using the methods suggested by Lipsey & Wilson (2001). If these statistics are also missing, the review authors will request information from the study investigators.

If missing summary data necessary for the calculation of effect sizes cannot be derived or retrieved, the study results will be reported in as much detail as possible, that is, the study will be included in the review but excluded from the meta‐analysis. If data is missing regarding moderators, we will use methods for multiple imputation in order to not bias our results by excluding these studies (see Rubin, 1996 and Pigott, 2009 for why leaving out studies/effect sizes with missing values normally yields biased estimates). We will use the Stata command *mi impute* with sequential imputation using chained equations to generate values for missing observations. All variables without missing observations will be used in the estimation to impute values for variables with missing observations.

#### 3.6.8 Assessment of heterogeneity

As the interventions deal with diverse populations of participants (both foster and adoptive children within a very large age range and adoptive children from different countries of origin), and we therefore expect heterogeneity among primary study outcomes, all analyses of the overall effect will be inverse variance weighted using random effects statistical models that incorporate both the sampling variance and between study variance components into the study level weights. Random effects weighted mean effect sizes will be calculated using 95% confidence intervals and we will provide a graphical display (forest plot) of effect sizes. Graphical displays for meta‐analysis performed on ratio scales sometimes use a log scale, as the confidence intervals then appear symmetric. This is however not the case for the software Revman 5, which we plan to use in this review^2^. The graphical displays using odds ratios and the mean effect size will be reported as a odds ratio. Heterogeneity among primary outcome studies will be assessed with *χ* ^2^ (Q) tests, and e *I*‐squared, and *τ*‐squared statistics (Higgins, Thompson, Deeks, & Altman, 2003). Any interpretation of *χ* ^2^ tests will be made cautiously on account of its low statistical power.

For subsequent analyses of moderator variables that may contribute to systematic variations, we will use the mixed‐effects regression model. This model is appropriate if a predictor explaining some between‐studies variation is available but there is a need to account for the remaining uncertainty (Hedges & Pigott, 2004; Konstantopoulos, 2006).

#### 3.6.9 Assessment of reporting biases

Reporting bias refers to both publication bias and selective reporting of outcome data and results. Here, we state how we will assess publication bias. We will use funnel plots for information about possible publication bias if we find sufficient studies (Higgins & Green, 2011). However, asymmetric funnel plots are not necessarily caused by publication bias (and publication bias does not necessarily cause asymmetry in a funnel plot). If asymmetry is present, we will consider possible reasons for this.

#### 3.6.10 Data synthesis

The proposed project will follow standard procedures for conducting systematic reviews using meta‐analysis techniques. The overall data synthesis will be conducted where effect sizes are available or can be calculated, and where studies are similar in terms of the outcome measured. Thus, we hope to be able to perform multiple random‐effects meta‐analyses based on standardised mean differences (*d* or Hedge's *g*). Meta‐analysis of outcomes will be conducted on each metric (conceptual oucomes as outlined in section “*Types of outcomes measures”*) separately. By conceptual outcome, we mean that we may choose to combine different measures if they measure the same or very similar underlying phenomena, such as children's psychological adjustment. When combining different measures of an underlying phenomenon, we will be transparent about the measures used in the included primary studies. As different computational methods may produce effect sizes that are not comparable, we will be transparent about all methods used in the primary studies (research design and statistical analysis strategies) and use caution when synthesising effect sizes. When effect sizes used in the data synthesis are odds ratios, they will be log transformed before being analysed. The reason is that ratio summary statistics all have the common features that the lowest value that they can take is 0, that the value 1 corresponds with no intervention effect, and that the highest value an odds ratio can ever take is infinity. This number scale is not symmetric. The log transformation makes the scale symmetric: the log of 0 is minus infinity, the log of 1 is zero, and the log of infinity is infinity. Studies that have been coded with a Critical risk of bias will not be included in the data synthesis.

We anticipate that some studies may provide results separated by for example age and/or adopted vs. foster children. We will include results for all groups. To take into account the dependence between such multiple effect sizes from the same study, we will apply robust variance estimation (RVE) approach (Hedges, Tipton, & Johnson, 2010). An important feature of this analysis is that the results are valid regardless of the weights used. For efficiency purposes, we will calculate the weights using a method proposed by Hedges et al (2010). This method assumes a simple random‐effects model in which study average effect sizes vary across studies (*τ* ^2^) and the effect sizes within each study are equicorrelated (*p*). The method is approximately efficient, since it uses approximate inverse‐variance weights: they are approximate given that *p* is, in fact, unknown and the correlation structure may be more complex. We will calculate weights using estimates of *τ* ^2^, setting *p* = 0.80 and conduct sensitivity tests using a variety of *p* values; to asses if the general results and estimates of the heterogeneity is robust to the choice of *p*. We will use the small sample adjustment to the residuals used in RVE as proposed by Bell and McCaffrey (2002) and extended by McCaffrey, Bell, and Botts (2001) and by Tipton (2015). We will use the Satterthwaite degrees of freedom (Satterthwaite, 1946) for tests as proposed by Bell and McCaffrey (2002) and extended by Tipton (2015). We will use the guidelines provided in Tanner‐Smith & Tipton (2014) to evaluate if there are enough studies for this method to consistently estimate the standard errors.

If there is not a sufficient number of studies to use RVE, we will conduct a data synthesis where we use a synthetic effect size (the average) in order to avoid dependence between effect sizes.

If there are a sufficient number of studies, we will apply the RVE approach and use approximately inverse variance weights calculated using a method proposed by Hedges et al. (2010). This technique calculates standard errors using an empirical estimate of the variance: it does not require any assumptions regarding the distribution of the effect size estimates. The assumptions that are required to meet the regularity conditions are minimal and generally met in practice. This more robust technique is beneficial because it takes into account the possible correlation between effect sizes separated by the covariates within the same study and allows all of the effect size estimates to be included in meta‐regression. We will calculate weights using estimates of *τ* ^2^, setting *p* = 0.80 and conduct sensitivity tests using a variety of *p* values; to asses if the general results is robust to the choice of *p*. We will use the small sample adjustment to the residuals used in RVE and the Satterthwaite degrees of freedom (Satterthwaite, 1946) for tests (Tipton, 2015). The results in Tipton (2015) suggests that the degrees of freedom depend on not only the number of studies but also on the type of covariates included in the meta‐regression. The degrees of freedom can be small, even when the number of studies is large if a covariate is highly unbalanced or a covariate with very high leverage is included, The degrees of freedom will vary from coefficient to coefficient. The corrections to the degrees of freedom enable us to assess when the RVE method performs well. As suggested by Tanner‐Smith & Tipton (2014) and Tipton (2015) if the degrees of freedom are smaller than four, the RVE results should not be trusted.

#### 3.6.11 Subgroup analysis and investigation of heterogeneity

We will investigate the following factors with the aim of explaining potential observed heterogeneity: study‐level summaries of participant characteristics (e.g., studies considering a specific population such as foster or adopted children, domestic vs. international adoptees, age or socioeconomic level or studies where separate effects for foster/adoptive families or low/high socioeconomic status are available), the duration of the intervention and the number of sessions, the format (single vs. multifamily) and possibly whether the intervention is based exlusively or only partly on attachment theory.

If the number of included studies is sufficient and given there is variation in the covariates, we will perform moderator analyses (multiple meta‐regression using the mixed model) to explore how observed variables are related to heterogeneity.

We will report 95% confidence intervals for regression parameters. We will estimate the correlations between the covariates and consider the possibility of confounding. Conclusions from meta‐regression analysis will be cautiously drawn and will not solely be based on significance tests. The magnitude of the coefficients and width of the confidence intervals will be taken into account as well. Otherwise, single factor subgroup analysis will be performed. The assessment of any difference between subgroups will be based on 95% confidence intervals. Interpretation of relationships will be cautious, as they are based on subdivision of studies and indirect comparisons.

In general, the strength of inference regarding differences in treatment effects among subgroups is controversial. However, making inferences about different effect sizes among subgroups on the basis of between‐study differences entails a higher risk compared to inferences made on the basis of within study differences; see Oxman & Guyatt (1992). We will therefore use within study differences where possible.

We will also consider the degree of consistence of differences, as making inferences about different effect sizes among subgroups entails a higher risk when the difference is not consistent within the studies; see Oxman & Guyatt (1992).

#### 3.6.12 Sensitivity analysis

Sensitivity analysis will be carried out by restricting the meta‐analysis to a subset of all studies included in the original meta‐analysis and will be used to evaluate whether the pooled effect sizes are robust across components of risk of bias. We will consider sensitivity analysis for each domain of the risk of bias checklists and restrict the analysis to studies with a low risk of bias. Sensitivity analyses with regard to research design and statistical analysis strategies in the primary studies will be an important element of the analysis to ensure that different methods produce consistent results.

#### 3.6.13 Treatment of qualitative research

We do not plan to include qualitative research.

## AUTHOR CONTRIBUTIONS

Nina Thorup Dalgaard is a psychologist, PhD Nina has clinical experience with parent/child attachment interventions and has published research focusing on attachment and children's psychosocial adjustment in refugee families as well as systematic reviews focusing on family violence and trauma communication in traumatised refugee families.

Morten Kjær Thomsen holds an MSc in Psychology and an MPhil in Social and Developmental Psychology (Cantab). Morten previously worked as a clinical psychologist in the Danish health care sector, doing psychological assessments and attachment‐focused therapy. Furthermore, Morten has experience from research‐positions in large‐scaled Danish and UK cohort studies. Morten is trained in the methodology of meta analytic reviews, including risk of bias assessment and advanced statistical analyses.

Maiken Pontoppidan, PhD (public health), MA (Psychology and History) is experienced in conducting randomised controlled trials and systematic reviews of evaluating the effects of parenting interventions for parents with young children. Maiken has published two systematic reviews on parenting interventions and one has recently been submitted for publication. Maiken is currently PI of two interventions projects examining the effects of mentalization based interventions for vulnerable pregnant women and is in the trial steering group of two intervention projects aimed at vulnerable families with infants.

### Systematic review methods

Trine Filges, PhD (economics): is an experienced systematic reviewer and methodologist, having completed a number of systematic reviews in social welfare topic areas as well as in the field of education. Trine has published thirteen Campbell Systematic reviews, is currently the lead reviewer on three Campbell Systematic Reviews, further involved as a reviewer in two Campbell Systematic Reviews and has published systematic and meta‐analytic reviews in high‐impact journals. Trine's fields of expertise are systematic review methods and statistical analysis; and she will contribute to the quantitative data extraction, methodological quality appraisal and meta‐analysis.

Nina Thorup Dalgaard (Please see description above)

Maiken Pontoppidan (Please see description above)

### Statistical analysis

Trine Filges (please see description above)

Morten Kjær Thomsen (please see description above)

### Information retrieval

Bjørn Christian Arleth Viinholt (information specialist): has 4 years of experience in developing and writing systematic reviews. As a part of undertaking systematic reviews, Bjørn has experience in developing systematic search strategies and processes of reference management. Bjørn will contribute with assisting and development of the systematic search strategy, executing the searches, and assist with reference management and grey literature searches. Bjørn will also assist with aspects relating to systematic literature searches in Campbell review methodology.

## Footnotes

^1^

http://www.europarl.europa.eu/RegData/etudes/BRIE/2016/583860/EPRS_BRI(2016)583860_EN.pdf

^2^
If we apply robust variance estimation, the analysis will be conducted in STATA or R as robust variance estimation is not implemented in Revman 5.

## APPENDIX A

### First and second level screening

First level screening is on the basis of titles and abstracts. Second level is on the basis of full text

Reference id. No.:
Reviewers initials:
Source:
Year of publication:
Country/countries of origin:
Author(s):

The study will be excluded if one or more of the answers to question 1–4 are “No.” If the answers to question 1 to 4 are “Yes” or “Uncertain,” then the full text of the study will be retrieved for second level eligibility. All unanswered questions need to be posed again on the basis of the full text. If not enough information is available, or if the study is unclear, the author of the study will be contacted if possible.

**Screening questions:**

1. Does the study focus on interventions offered to either foster/adoptive families?
  Yes – include
  No – if no then stop here and exclude
  Uncertain – include
  Question 1 guidance:
  The population of this review are foster and adoptive families. Studies focusing on interventions for at risk families more broadly will not be eligible.
2. Are the participants both parents and children in foster and/or adoptive families?
  Yes – include
  No – if no then stop here and exclude
  Uncertain – include
  Question 2 guidance:
  The intervention must involve the direct participation of at least one parent and at least one child aged between 0 and 17 years at the beginning of the intervention in each family. Studies focusing on parental counselling, parental support groups or individual psychotherapy for children or adults will not be eligible.
3. Is the intervention at least partly be informed by attachment theory? (this may be difficult to determine based on the title and abstract alone)
  Yes – include
  No – if no then stop here and exclude
  Uncertain – include
  Question 3 guidance:
  In order to be eligible for the review, the description of the intervention must make explicit reference to attachment theory and the treatment goals must involve at least one of the following: an increase in attachment security, a decrease in disorganised attachment, an increase in parental sensitivity, an increase in parental emotional availability, an increase in positive dyadic interaction or an increase in the psychosocial adjustment of the child. Interventions based on an eclectic theoretical approach may be eligible if attachment theory is part of the theoretical framework.
4. Is the report/article a quantitative evaluation study with a comparison condition? If not, does the paper make reference to a normative sample for comparison of attachment distributions preintervention and postintervention?

Yes – include

No – if no then stop here and exclude

Uncertain – include

Question 4 guidance:

We are only interested in primary quantitative studies with a comparison group or where a distribution of attachment categories is compared with a normative non‐clinical sample, where the authors have analysed the data. We are not interested in theoretical papers on the topic or surveys/reviews of studies of the topic. (This question may be difficult to answer on the base of titles and abstracts alone).

### Data extraction

**Table:**

**Names of author(s)**
**Title**
**Language**
**Journal**
**Year**
**Country**
**Type of families (foster/adoptive or both)**
**Participant characteristic (child age at adoption and at the intervention, gender, socioeconomic status, ethnicity, child country of origin)**
**Programme feature** *Format, one family vs. multiple family therapy*
**Programme feature** *Content*, code yes if the intervention was based exclusively on attachment theory
**Programme feature** *Participants, only one parent and child in each family, vs. more family members*
**Programme feature** *Therapist characteristics, educational background, years of experience*
**Programme feature** *Duration* (total duration, number of sessions and duration of sessions**)**
**Programme feature** *Setting (in the families’ homes, hospital, outpatient clinic, community facility)*
**Type of data used in study (independent observation, questionnaire, other (specify))**
**Level of aggregation (individual and/or family/dyad)**
**Time period covered by analysis (divide into intervention and follow up)**
**Sample size (divide into treated/comparison)**

**Outcome measures**

Instructions: Please enter outcome measures in the order in which they are described in the report. Note that a single outcome measure can be completed by multiple sources and at multiple points in time (data from specific sources and time‐points will be entered later).

**Table:**

#	Outcome & measure	Reliability & Validity	Format	Direction	Pg# & notes
1		Info from: Other samples This sample Unclear Info provided:	Dichotomy Continuous	High score or event is Positive Negative Can't tell	

*Repeat as needed

**OUTCOME DATA**

**DICHOTOMOUS OUTCOME DATA**

**Table:**

OUTCOME	TIME POINT (s) (record exact time from participation, there may be more than one, record them all)	SOURCE	VALID Ns	CASES	NON‐CASES	STATISTICS	Pg. # & NOTES
		Questionnaire Admin data Other (specify) Unclear	Participation	Participation	Participation	RR (risk ratio) OR (odds ratio) SE (standard error) 95% CI DF *p*‐value (enter exact *p*‐value if available) *χ* ^2^ Other	
		
			Comparison	Comparison	Comparison		
		

Repeat as needed

**CONTINUOUS OUTCOME DATA**

**Table:**

OUTCOME	TIME POINT (s) (record exact time from participation, there may be more than one, record them all)	SOURCE (specify)	VALID Ns	Means	SDs	STATISTICS	Pg. # & NOTES
		Questionnaire Admin data Other (specify) Unclear	Participation	Participation	Participation	P t F Df ES Other	
		
			Comparison	Comparison	Comparison		
		

*Repeat as needed

### Assessment of risk of bias in included studies

#### User guide for unobservables

Systematic baseline differences between groups can compromise comparability between groups. Baseline differences can be observable (e.g., age and gender) and unobservable (to the researcher; e.g. motivation and “ability”). There is no single non‐randomised study design that always solves the selection problem. Different designs solve the selection problem under different assumptions and require different types of data. Especially how different designs deal with selection on unobservables varies. The “right” method depends on the model generating participation, that is, assumptions about the nature of the process by which participants are selected into a programme.

As there is no universal correct way to construct counterfactuals we will assess the extent to which the identifying assumptions (the assumption that makes it possible to identify the counterfactual) are explained and discussed (preferably the authors should make an effort to justify their choice of method). We will look for evidence that authors using, for example (this is NOT an exhaustive list):

**Natural experiments:**

Discuss whether they face a truly random allocation of participants and that there is no change of behaviour in anticipation of, for example, policy rules.

**Matching (including propensity scores):**

Explain and discuss the assumption that there is no selection on unobservables, only selection on observables.

**(Multivariate, multiple) Regression:**

Explain and discuss the assumption that there is no selection on unobservables, only selection on observables. Further discuss the extent to which they compare comparable people.

**Regression discontinuity:**

Explain and discuss the assumption that there is a (strict!) RD treatment rule. It must not be changeable by the agent in an effort to obtain or avoid treatment. Continuity in the expected impact at the discontinuity is required.

**Difference‐in‐difference (Treatment‐control‐before‐after):**

Explain and discuss the assumption that the trends in treatment and control groups would have been parallel, had the treatment not occurred.
